# Supplementary material for: How epigenome drives chromatin folding and dynamics, insights from efficient coarse-grained models of chromosomes
Source: PLoS Comput Biol. 2018 May 29;14(5):e1006159. doi: 10.1371/journal.pcbi.1006159 (PMC6003694; doi:10.1371/journal.pcbi.1006159)
Supplement: S1 Text — (PDF) [file pcbi.1006159.s001.pdf]

Supplementary Information

How epigenome drives chromatin folding and dynamics, insights from efficient coarse-grained models of chromosomes

Surya K. Ghosh* and Daniel Jost†
[1] *Univ Grenoble Alpes, CNRS, Grenoble INP, TIMC-IMAG, F-38000 Grenoble, France*

* Corresponding authors; surya.ghosh@univ-grenoble-alpes.fr
† Corresponding authors; daniel.jost@univ-grenoble-alpes.fr

1. SUPPLEMENTARY FIGURES

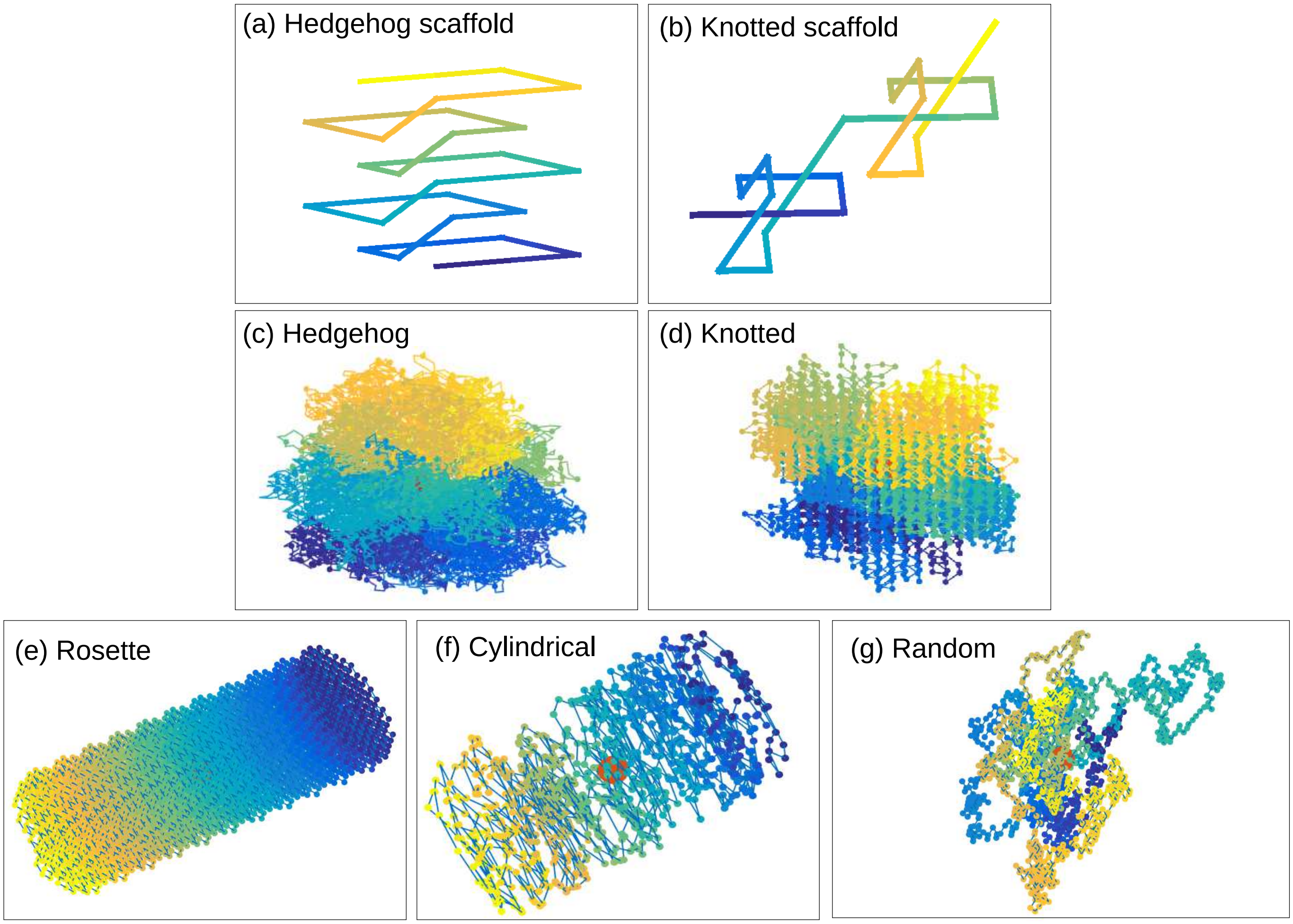

Figure A. Many types of distinctly different initial configurations are used to verify our simulation strategy. In (a) we show a small section of the central scaffold of the *unknotted* hedgehog structure and in (c) the corresponding full unknotted configuration. Similarly, in (b), a small portion of the *knotted* central scaffold is shown and the corresponding full configurations with the fixed number of knots is shown in (d). In the bottom panel, we show the full configuration of Rosette, Cylindrical and Random structure in (e),(f), and (g) respectively. The gradient of the color changes along the contour of the polymer and the big red dot in the middle of the whole configurations, represents the center of mass of the polymer. To construct an unknotted structure, we start with a helical structure with a much smaller number of monomer $N_0 << N$ at the center of the lattice. Because of the helical basis of N_0 beads as the central scaffold, the structure remains unknotted. To construct the knotted configuration, we started with a knotted configuration of length N_0 which contains a fixed number of knots. Around this knotted configuration we build our whole initial configuration of length N.

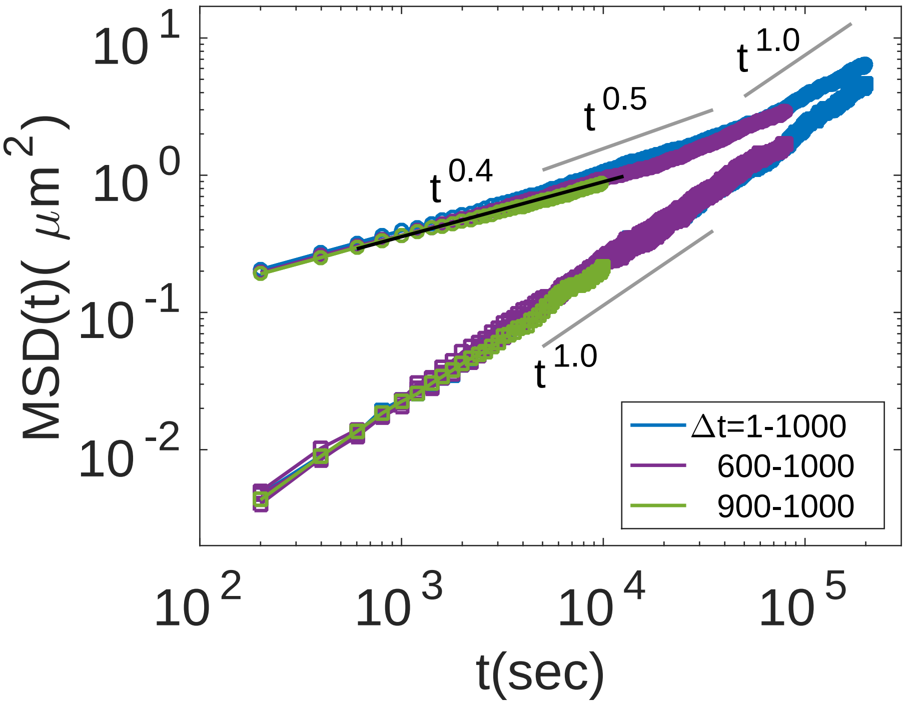

Figure B. The time evolution of the mean squared displacement $MSD(t)$ of the individual monomer g_1 (upper curve), and the $MSD(t)$ of the center of mass g_3 (lower curve) for the drosophila case ($L/L_e = 70$, $\rho = 0.009bp/nm^3$) at coarse graining $CG = 10$ kbp and Kuhn size of $N_k = 23$ kbp. We vary the measurement time window Δt and represented the corresponding MSDs where each unit of Δt represents 10^4 monte carlo steps. At steady state we perfectly recovered the $g_1 \sim t^{0.4}$ scaling. The center of mass MSD exhibits $g_3 \sim t^1$ scaling behaviour for any time window.

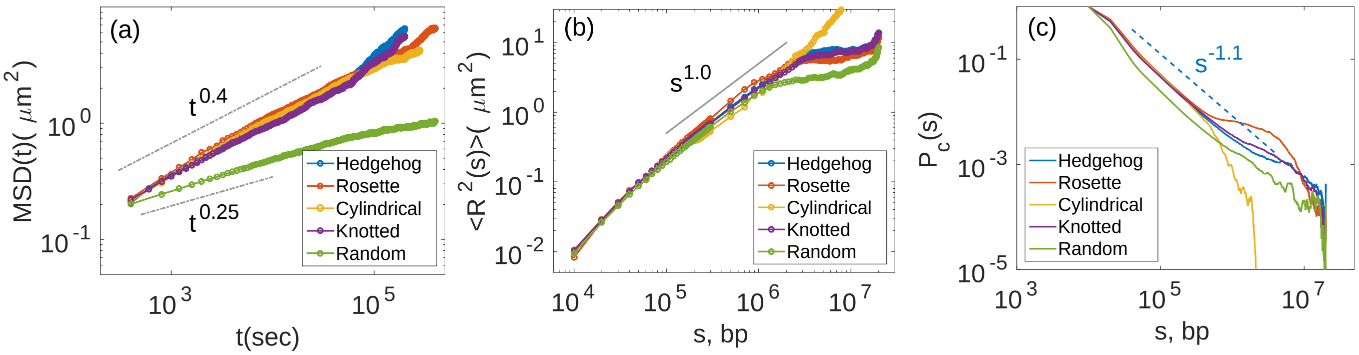

Figure C. Comparison between different types of initial configurations for the drosophila case ($L/L_e = 70$, $\rho = 0.009bp/nm^3$) at coarse-graining of $CG = 10$ kbp and Kuhn size $N_k = 23$ kbp. We have tested our simulation scheme with many initial knotted and unknotted configurations: hedgehog, rosette, cylindrical, knotted, and random. We recovered the $t^{0.4}$ scaling in g_1 for all configurations except the random initial configuration. For randomly knotted configuration, the polymer is entangled into itself and exhibits reptile motion with $g_1 \sim t^{0.25}$. We also observed $\langle R^2(s) \rangle \sim s^1$ for average physical distance squared and $P_c \sim s^{-1.1}$ scaling behavior for contact probability.

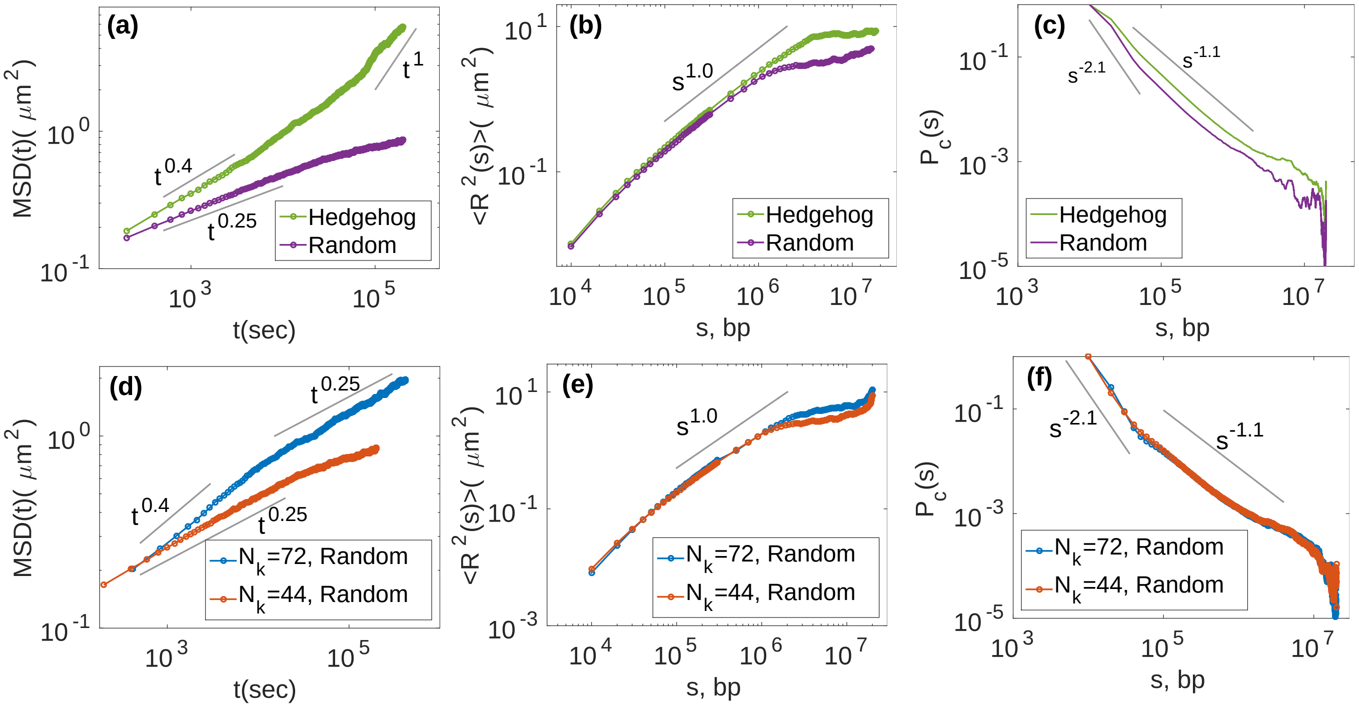

Figure D. Comparison between knot-free (hedgehog) and random configurations for the drosophila case ($L/L_e = 70$, $\rho = 0.009 bp/nm^3$). In the top panel, we compared the random configuration model with hedgehog configurations at fixed Kuhn size ($N_k = 44$ kbp) and coarse-graining (CG=10 kbp). In the bottom panel, we compared physical parameters of different N_k for random configurations. (a,d) individual MSD $g_1(t)$ as a function of time, (b,e) the average physical distance squared $\langle R^2(s)\rangle$ between any two monomers as a function of genomic distance s, (c,f) average contact probability $P_c(s)$. Simulation data confirmed that the motion of the chain significantly decreased due to the presence of knots. At small $N_k = 44$ kbp which correspond to higher lattice volumic fraction, we observed $g_1 \sim t^{0.25}$ scaling which is expected from an entangled polymer exhibiting reptile motion. The contact probability P_c, at short length scale of $s < 100$ kbp, scales as a self-avoiding random walk ($P_c \sim s^{-2.1}$). At intermediate and large length scale limit, 0.1 $Mbp < s <$ 10 Mbp we observed $s^{-1.1}$ (f). We conclude that our simulation strategy is general and worked for both random (knotted) and unknotted configurations.

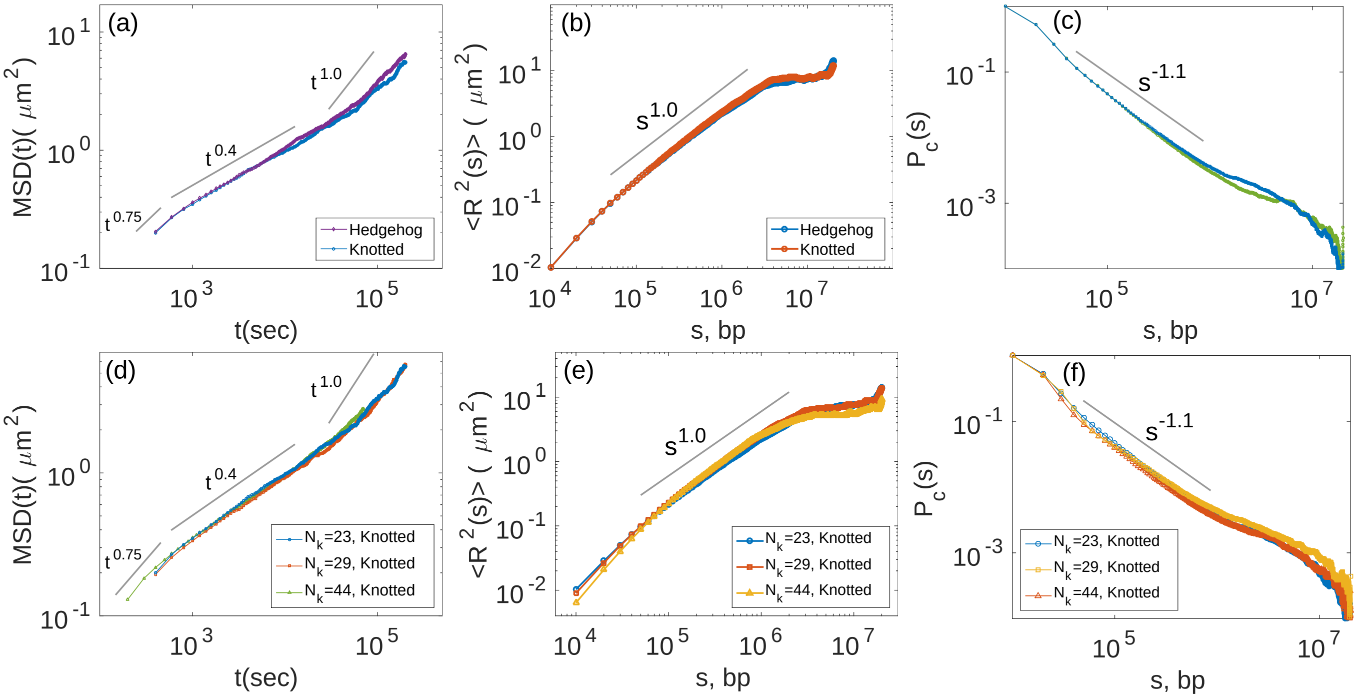

Figure E. Comparison between knot-free (hedgehog) and configurations with a fixed number of knots for the drosophila case ($L/L_e = 70$, $\rho = 0.009bp/nm^3$). In the top panel, we compared the knotted configuration model with hedgehog configurations at fixed Kuhn size ($N_k = 23$ kbp) and coarse-graining ($CG = 10$ kbp). In the bottom panel, we compared physical parameters of different N_k for knotted configurations. (a,d) individual MSD $g_1(t)$ as a function of time, (b,e) the average physical distance squared $\langle R^2(s)\rangle$ between any two monomers as a function of genomic distance s, (c,f) average contact probability $P_c(s)$. From the simulation data, we confirm that the presence of few knots does not change the structural and dynamics properties significantly. At any Kuhn size, we observed $g_1 \sim t^{0.4}$ scaling in the intermediate time and initially at short timescale, due to the lattice effect, we observed $\sim t^{0.75}$. The contact probability P_c, at intermediate and large length scale limit, we observed $P_c(s) \sim s^{1.1}$ (f), which is similar to the experimentally observed value. We conclude that our simulation strategy is general and worked for both knotted and unknotted configurations.

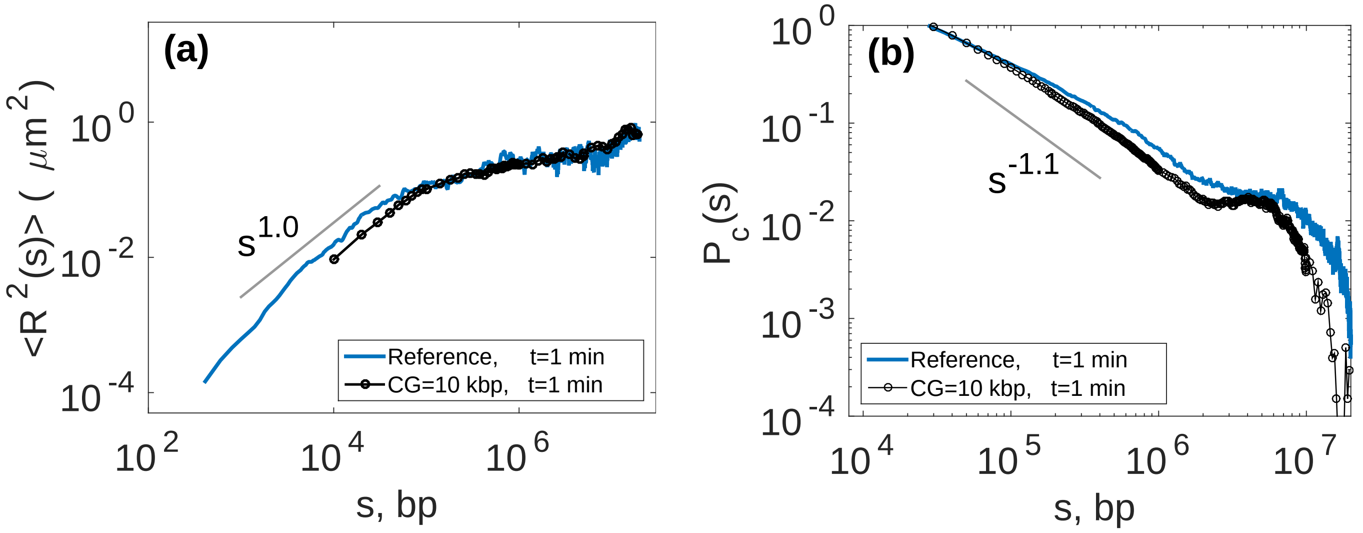

Figure F. We compared different physical properties between reference model and 10 kbp coarse graining at short time ($t = 1\ min$) for the drosophila case ($L/L_e = 70$, $\rho = 0.009bp/nm^3$). The mean squared distance between any to monomer $\langle R^2\rangle(s)$ is represented in (a) and the average contact probability $P_c(s)$ is represented in (b). While initial configurations are quite similar regarding $\langle R^2\rangle$, they differ strongly at long range for P_c.

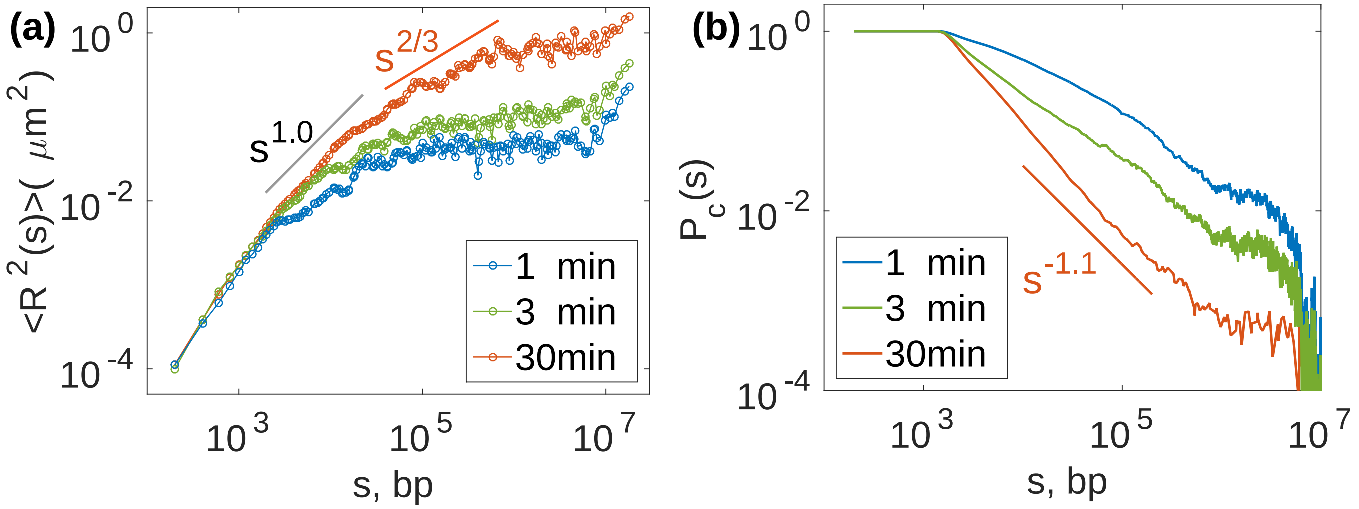

Figure G. The reference model of drosophila chromosome ($L/L_e = 70$, $\rho = 0.009 bp/nm^3$). The time evolution of the physical distance between any two genomic loci $\langle R^2 \rangle$ in (a) and the average contact probability $P_c(s)$ at coarse graining of $CG = 0.2$ kbp and $N_k = 1$ kbp. Because of the huge computational time we were able to study just up to 30 min of real time. We approximately extracted the characteristic scaling behavior of drosophila chromosome: $\langle R^2 \rangle \sim s^{2/3}$, $P_c \sim s^{-1.1}$.

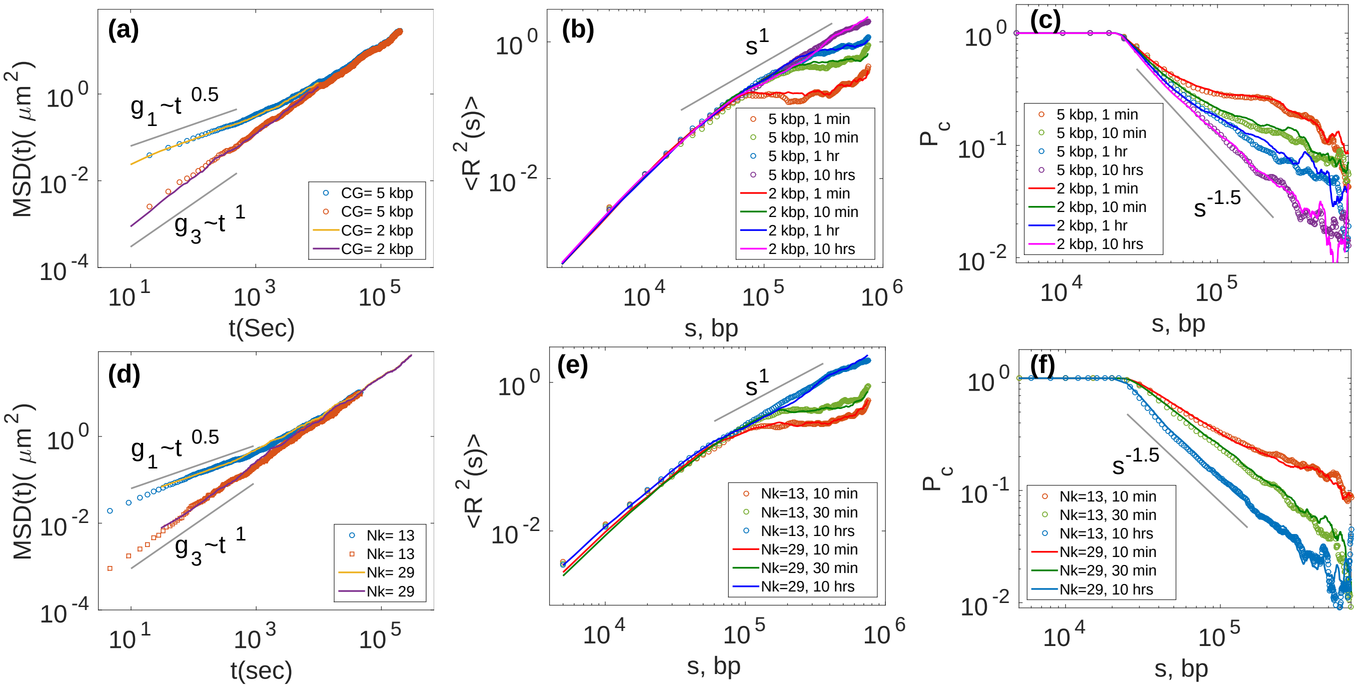

Figure H. Physical properties of yeast chromosome ($L/L_e = 0.8$, $\rho = 0.005 bp/nm^3$). In the top panel, the exactly same time evolution of physical properties are observed as we change coarse graining $CG = 2,\ 5$ kbp, at fixed Kuhn size of $N_k = 20$ kbp. The bottom panel represents, the time evolution of physical properties for different Kuhn size ($N_k = 13,\ 29$ kbp) at a fixed coarse graining of $CG = 5$ kbp. (a) Mean squared displacement of individual monomers g_1 is plotted as the top curve and that of the center of mass g_3 is represented as the bottom curve. (b) Time evolution of the mean squared distance $\langle R^2(s) \rangle$ between any pairs of monomers separated by a genomic distance s. (c) Time evolution of averaged contact probability $P_c(s)$. In the bottom panel, the time evolution of physical properties is represented as we vary $N_k = 13,\ 29$ kbp at fixed coarse graining of $CG = 5$ kbp. (d) $g_1(t)$ (top curves) and $g_3(t)$ (bottom curves) after time mapping, (e) $\langle R^2 \rangle$ and (f) $P_c(s)$. At short time $g_1 \sim t^{0.5}$ and at steady state we have $g_1 \sim g_3 \sim t^1$, $\langle R^2 \rangle \sim s^1$ and $P_c(s) \sim s^{-1.5}$. For simulations parameters see Table.A.

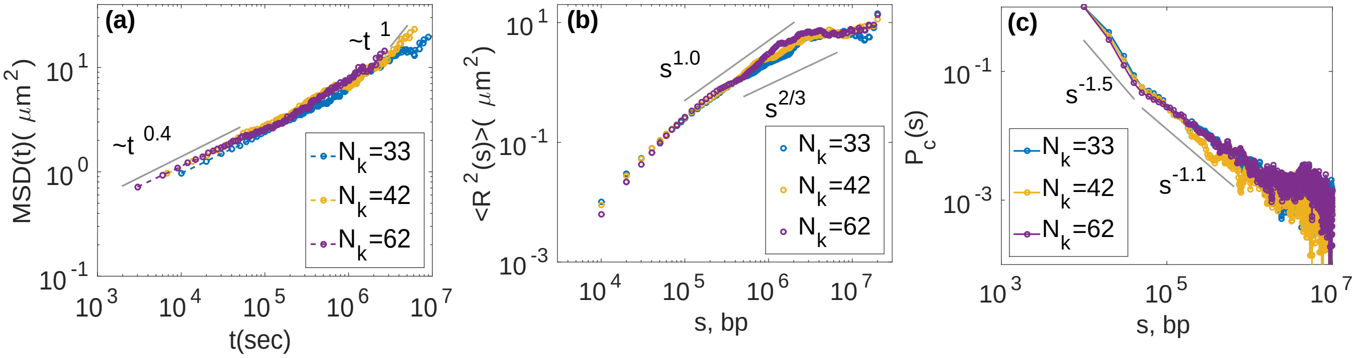

Figure I. Physical properties of mammalian chromosomes ($L/L_e = 200$, $\rho = 0.015bp/nm^3$) at coarse graining $CG = 10$ kbp for different Kuhn sizes $N_k = 33,\ 42,\ 62$ kbp. (a) Mean squared displacement g_1 for various Kuhn sizes. (b) Average end to end distance $\langle R^2 \rangle$ as a function of genomic distance s. (c) Average contact probability P_c as a function s. $\langle R^2 \rangle$, P_c are calculated from the configurations collected in the 1 to 10 hrs time window. For simulations parameters see Table.B.

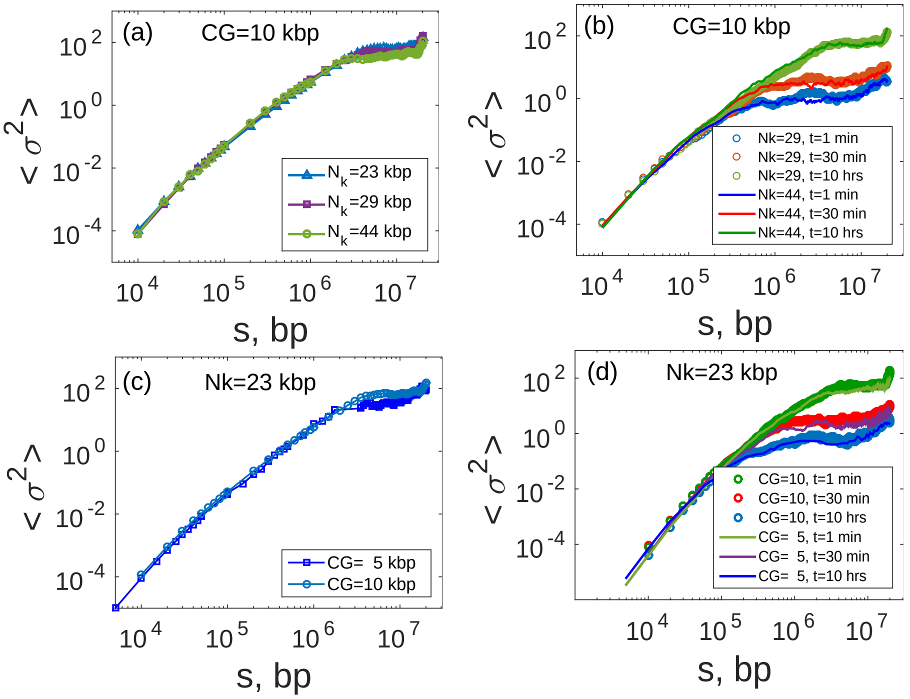

Figure J. The average second moment ($\langle \sigma^2 \rangle$) of the square of the distance (R^2) between any two monomers for the drosophila case ($L/L_e = 70$, $\rho = 0.009bp/nm^3$). In the top panel, we observed a perfect matching in steady state (a) and time evolution (b) for $\langle \sigma^2 \rangle$ at different N_k for the fixed coarse graining of $CG = 10$ kbp. In the bottom panel, for a fixed N_k we compared the steady state (c) and dynamic (d) properties of $\langle \sigma^2 \rangle$ at different coarse graining.

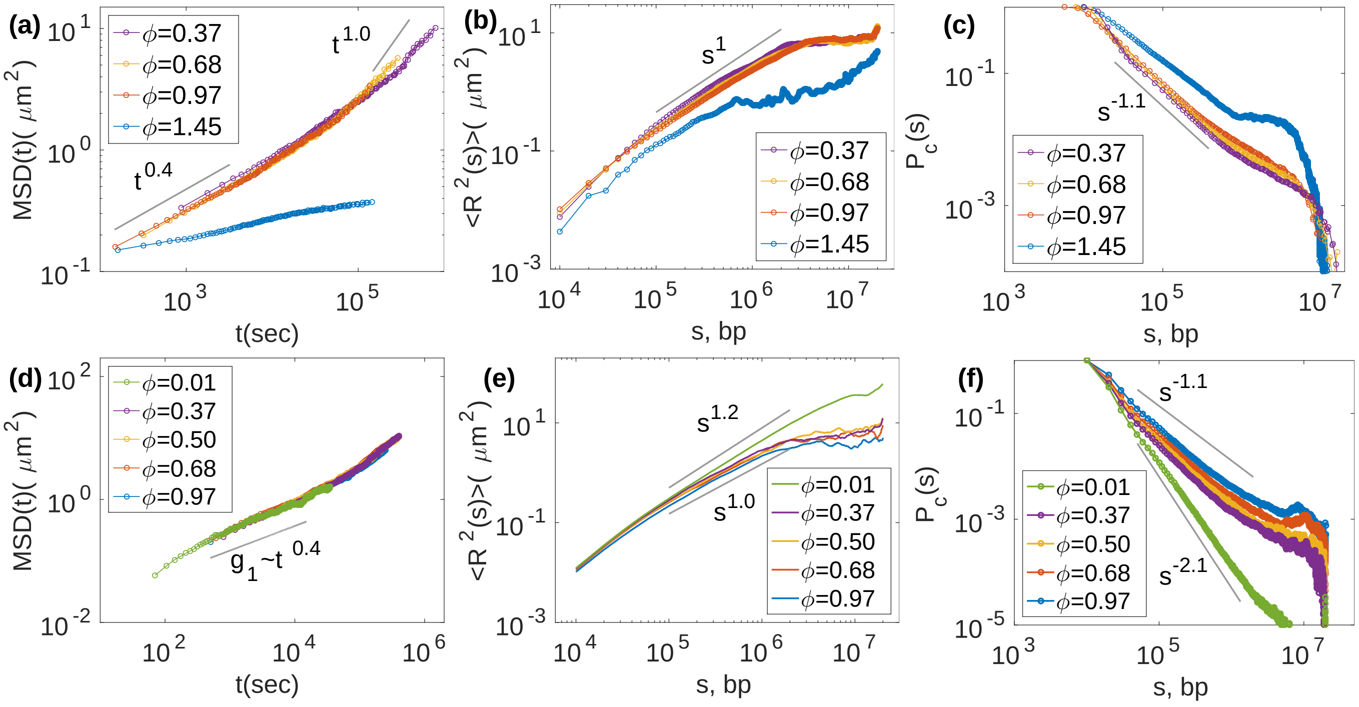

Figure K. Effect of lattice volumic fraction Φ on physical properties of lattice polymer model for drosophila at $CG = 10$ kbp. In the top panel, we vary Φ such a way that the base pair density (ρ) and entanglement regime (L/L_e) are conserved. In the bottom panel, we change Φ arbitrarily, keeping other simulation parameters constant ($N_k = 23$ kbp, $\phi = 0.97$), as a result, we did not take into account the preservation of the actual L/L_e value. (a,d) MSDs of individual monomer g_1, (b,e) the physical distance between any two genomic loci $\langle R^2 \rangle$ and (c,f) the average contact probability $P_c(s)$. In the top panels, up to $\Phi = 1$ all the physical properties calculated from simulations represent the true nature of the system, same as the reference model. At $\Phi > 1$, dynamics gets restricted due to very high lattice volumic fraction and $\langle R^2 \rangle$ and P_c start deviating from the actual results. For example, $P_c(s)$ shows slow decay, close to the initial configuration. In the bottom panel, changing the lattice volumic fraction influences strongly $\langle R^2 \rangle$ and $P_c(s)$ at all length scales. At extremely low density the chain behaves like a self-avoiding random walk, and we recover the scaling of $P_c(s) \sim s^{-2.1}$ and $\langle R^2 \rangle \sim s^1$. From this study, we conclude that the importance of choosing simulation parameter Φ such a way that it preserves the true physical properties of the system. We also realized that our strategy is not accurate for $\Phi > 1$.

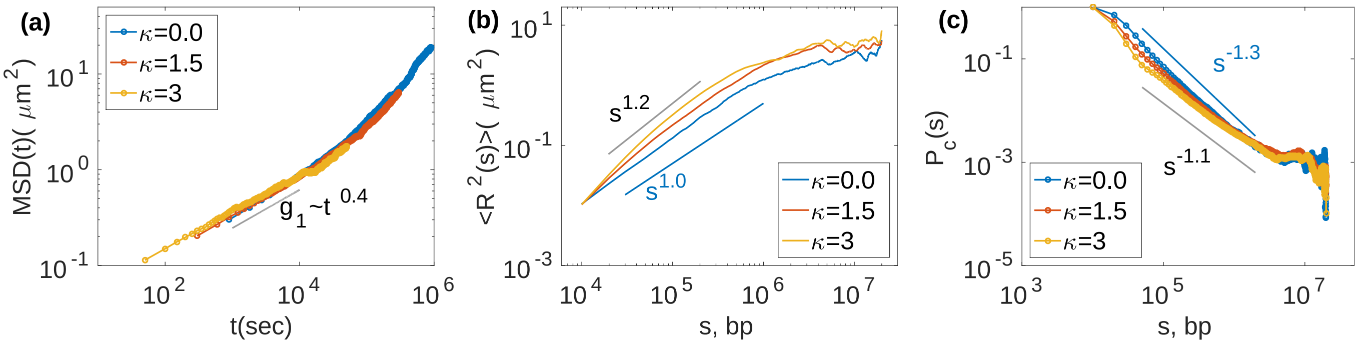

Figure L. Effect of bending rigidity κ on different physical properties ($CG = 10$ kbp, $\Phi = 0.97$). We vary κ arbitrarily, keeping other simulation parameters constant, as a result, we did not take into account the preservation of the actual L/L_e value. (a) The effect of κ is adjusted through time mapping, as different κ correspond to different time mapping. (b,c) show the effect of κ on $\langle R^2 \rangle$ (b) and on P_c (c). At small length scale the effect of κ is much stronger, where as at large length scale $s > 1$ *mbp*, the effect is negligible when the chain is already decorrelated. At short length scale for stronger κ we have smaller P_c with faster decay, and we have larger $\langle R^2 \rangle$ corresponding to a bigger overall size of the chain. Stiff bending constant leads to more rambling configurations.

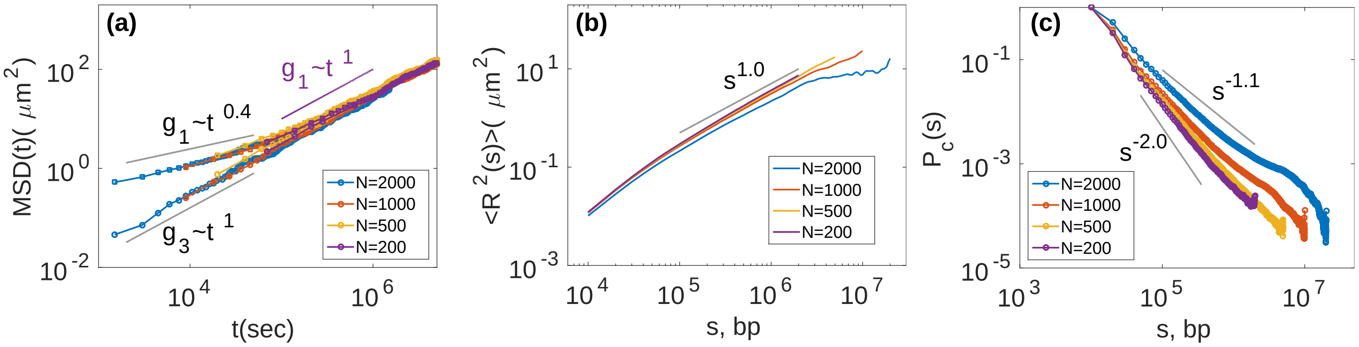

Figure M. Effect of simulating subchains instead of the full polymer. (a) Mean squared displacement of individual monomers g_1, center of mass g_3, (b) physical distance squared $\langle R^2 \rangle$ between any two monomers separated by a genomic distance s, and (c) the contact probability P_c, are represented for different chain lengths N. As we decrease chain length, the polymers behave as an isolated chain and reach steady state very quickly. Similarly for smallest N, we observed $\langle R^2 \rangle \sim s^1$ and $P_c(s) \sim s^{-2.0}$, which are close to the self-avoiding freely jointed chain limit.

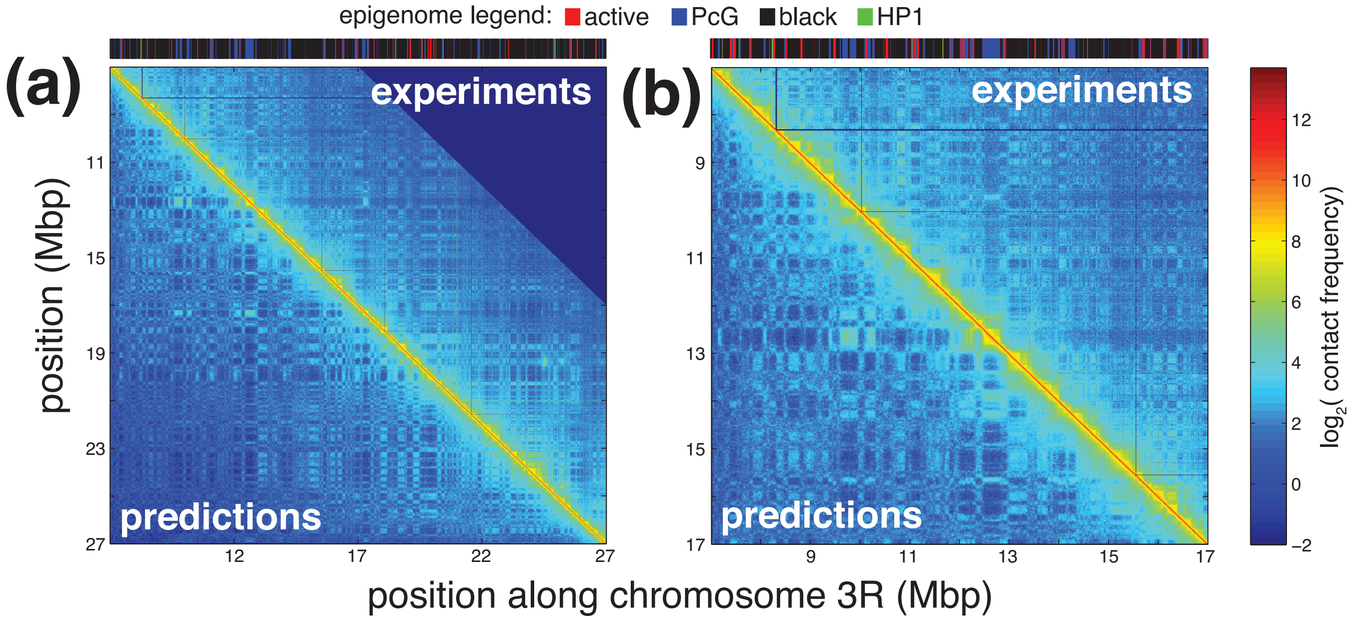

Figure N. (a) Experimental Hi-C map for the investagated 20 Mbp region of chromosome 3R. Corresponding epigenome is shown on top. (b) Predicted ($E_i = -0.1\ kT$) vs experimental contact maps for a 10 Mbp region. Predicted data were multiplied by a factor 2500 to adjust scale with experiments.

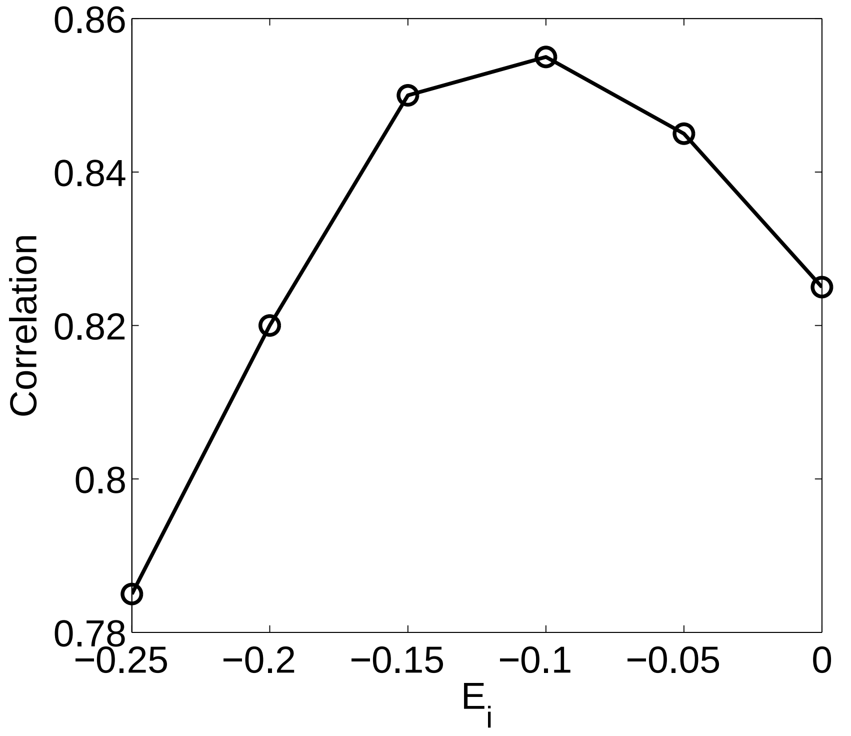

Figure O. Pearson correlation between the predicted and experimental Hi-C data as a function of the interaction strength E_i.

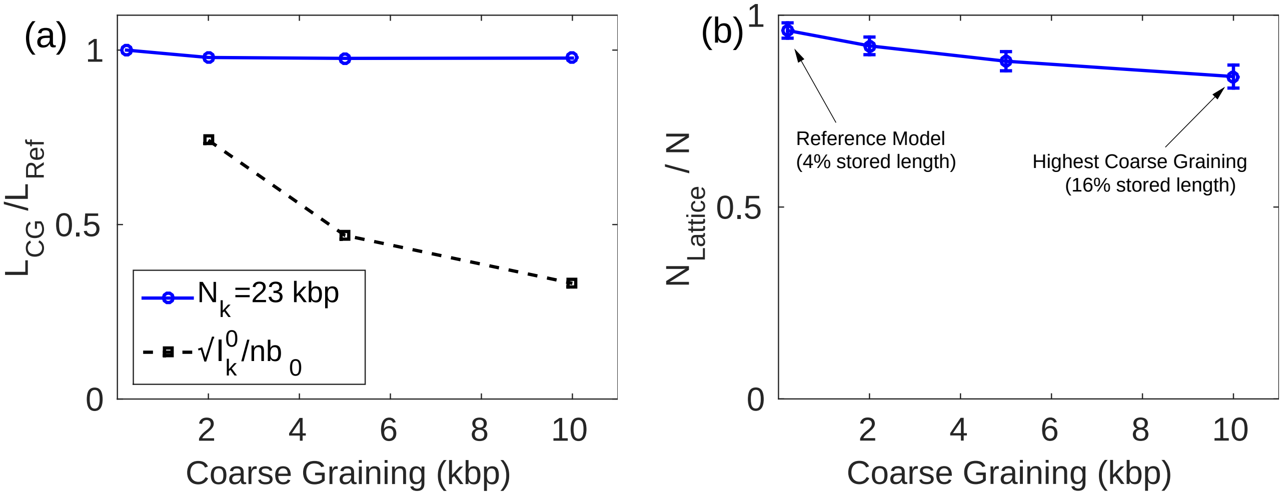

Figure P. (a) For a fixed Kuhn size $N_k = 23$ kbp, we compared the contour length of the coarse-grained polymer ($L_{CG} = Nb$) with the reference model ($L_{Ref} = N_0 b_0$). The ratio does not depend strongly on the coarse-graining for a fixed Kuhn size. Alternatively by choosing the CG bead size as the mean end to end distance of the corresponding fine scale model, the deformation ($\sqrt{l_k^0/nb_0}$) is stronger (dotted line). (b) Comparison of the polymer size (N monomers) with the "simulated" lattice polymer size $N_{Lattice}$ defined as the number of lattice sites actually occupied by the polymer (due to double occupancy $N_{Lattice} \leq N$). For example, in the reference model, where the lattice density is quite low $\phi \approx 0.04$, we still have 4% of it's length stored in the form of double occupancy.

2. SUPPLEMENTARY TABLES

2.1. Simulation parameters for yeast chromosome

**200 bp**				**2 kbp**				**5 kbp**			
Φ_0	N_k^0	b_0	l_k^0	Φ	N_k	b	l_k	Φ	N_k	b	l_k
0.023	1.0	11.0	56.4	0.016	29.2	20.8	304	0.11	27	53.6	292
				0.027	20.1	25.1	250	0.17	20	62.5	250
				0.054	13.0	31.3	198	0.30	13	75.1	209
				0.093	9.10	37.5	166	0.58	8.9	93.9	165

Table A. Table for the simulation parameters of yeast chromosome at different coarse-graining of CG = 0.2 kbp (reference model), 2 kbp, and 5 kbp. Here Φ is the lattice volumic fraction, N_k is the Kuhn size in kbp, b is the bead size in nm and l_k is the Kuhn length in nm. The corresponding physical parameters associated to reference model are Φ_0, N_k^0, b_0 and l_k^0. The general characteristics of yeast chromosome we used here are length $L = 750$ kbp, base pair density $\rho = 0.005\ bp/nm^3$ and the corresponding entanglement length we calculated $L_e = 924.2$ kbp.

2.2. Simulation parameters for mammalian chromosome

**200 bp**				**2 kbp**				**5 kbp**				**10 kbp**			
Φ_0	N_k^0	b_0	l_k^0	Φ	N_k	b	l_k	Φ	N_k	b	l_k	Φ	N_k	b	l_k
0.043	1.0	10.6	55.4	0.021	49	15.8	389	0.09	62	35.4	434	0.37	62	70.7	448
				0.027	42	17.3	364	0.17	42	43.2	364	0.68	42	86.4	354
				0.039	33	19.4	322	0.24	33	48.6	298	0.97	33	97.2	323

Table B. Table for simulation parameters for the mammalian chromosome at different coarse-graining: 200 bp (reference fine-scale model), 2, 5 and 10 kbp. Here lattice volumic fraction Φ, Kuhn size $N_K \equiv l_k/b$ (in kbp-unit), nearest-neighbor distance b (in nm) and Kuhn length l_k (in nm). The corresponding physical parameters associated to reference model are Φ_0, N_k^0, b_0 and l_k^0. We simulated a portion of mammalian chromosome of length 20 Mbp with base pair density $\rho = 0.015\ bp/nm^3$ and the corresponding entanglement length, we have $L_e = 102.7$ kbp.

2.3. Chromatin state positioning along drosophila chromosome 3R

	Position	State
1	9.70	PcG
2	11.1	Active
3	12.24	PcG
4	12.64	PcG
5	13.04	Black
6	15.44	Black
7	21.88	PcG
8	23.3	Black

Table C. Position along chromosome 3R and corresponding chromatin state (PcG: "blue" state, Black: "black" state, Active: "red" state in the epigenome legend of Fig. 6.) for the 8 loci tracked with high precision in Sec.II.D.3 of the main text. Fig. 7c,d were computed from the relative distances between loci 1&4, 1&7, 2&8, 3 & 4, 4&5, 4&6.
